# Special issue for Thomas Scheper's retirement

**DOI:** 10.1002/elsc.202200004

**Published:** 2022-02-17

Biotechnology today has found its way into all industrial areas and is considered a major cornerstone, a vital tool belt for enabling a sustainable and environmentally friendly production matching the sustainable development goals (SDGs) of the United Nations and thus paving humanity's way to a sustainable and green future. The application spectrum is so broad that the amount of knowledge and information generated seems to exceed the capability of a single person to keep track of all areas of the ongoing research.

Thomas Scheper, to whom this special issue of *Engineering in Life Science* is dedicated, is the exception to this rule, as he is one of the last scientists in the field of biotechnology in Germany, who is able to provide active expertise in all the respective fields of research. He learned in Karl Schügerls group, who was the godfather of biotechnology in Germany and initiated the use of biological systems for technical processes and who always pleaded for a holistic view on biotechnology. With this mindset, Thomas Scheper took over the Professorship for Technical Chemistry and the Directorship of the renowned Institute of Technical Chemistry at the Leibniz University of Hannover from Schügerl in 1995 and continued the biotechnological research in all thinkable areas ranging from monument conservation to industrial process development. His major assets were always his creativity, his progressivity, his communication skills and his attitude to never give up on a promising topic. And so as the years went by he developed technologies, methods and instruments to enable robust biotechnological processes and novel industrial applications. Examples of his research group developments range from novel bioanalytical tools like 2D‐fluorescence spectrometers and in situ microscopy to reactor systems for human tissue cultivation, which have all by now found their way into everyday utilization and are nowadays considered state of the art.

Fellow researchers, who have worked together with Thomas Scheper on various topics in the past, contributed all articles in this special issue. For this reason, the research shown here is characterized by the same diversity of topics and interests, which he himself always displayed in his works. The special issue is released on the occasion of Thomas Scheper's retirement in 2022, which coincides with his 66th birthday. Referring to a famous song, this is actually when life really starts, and so we wish Thomas Scheper a happy start into the life‐after‐work and hope that he will have some entertaining time while reading the articles from all his former colleagues and fellows.

Your TCI

